# Diagnostic Sensitivity of Different Reference Bodies When Using Scheimpflug Tomography in a Myopic Population with Keratoconus

**DOI:** 10.1155/2019/2593404

**Published:** 2019-07-18

**Authors:** Daniel Garcerant, Ignacio Jiménez-Alfaro, Nicolás Alejandre

**Affiliations:** Fundación Jiménez Díaz, Madrid, Spain

## Abstract

**Purpose:**

To establish which reference body offers the greatest sensitivity in keratoconus (KC) diagnosis, obtain normative data for the myopic population with toric ellipsoid reference bodies, and determine the cutoff points for a population with KC.

**Methods:**

A retrospective, observational study of the entire Scheimpflug tomographer database of the Fundación Jiménez Díaz in Madrid was conducted to identify a normal myopic and a KC myopic population. Three different reference bodies were tested on all patients: best fit sphere (BFS), best fit toric ellipsoid with fixed eccentricity (BFTEFE), and best fit toric ellipsoid (BFTE). Anterior and posterior elevation measurements at the apex and thinnest point were recorded, as well as the root mean square of posterior elevations (RMS-P). Normative data were extracted, and receiver operating characteristic (ROC) curves were generated to obtain cutoff points between the normal and KC population.

**Results:**

A total of 301 eyes were included, comprising 219 normal myopic and 82 myopic KC eyes. BFS and BFTEFE produced the best results when measuring posterior elevation at the thinnest point. BFTE had better sensitivity with the RMS-P. From all measurements, best sensitivity (100%) was achieved with a cutoff point of 8 *μ*m of posterior elevation at the thinnest point using the BFTEFE. BFTE was found to hide the cone in certain patients.

**Conclusions:**

Posterior elevation measured at the thinnest point with a BFTEFE is the best-performing parameter and, therefore, is recommended to discriminate between normal and KC patients within a myopic population.

## 1. Introduction

Keratoconus is a bilateral, asymmetric, and progressively degenerative disease. Due to the gradual thinning and steepening of the cornea caused by this illness, patients experience increasing irregular astigmatism, which decreases visual acuity [1, 2]. The impact that this disease has on the quality of life can be significant, and as lost vision is difficult to regain, early detection is essential for proper follow-up and treatment [3, 4].

This early detection becomes even more important in patients undergoing laser refractive surgery. Laser procedures performed on individuals with subclinical and otherwise stable disease can cause these patients to enter the progressive stage [5–8]. As a result, this population requires the most sensitive screening.

Changes in posterior elevation have been described as one of the first detectable alterations in patients with keratoconus [9–11]. In addition, the root mean square (RMS) of elevation values is believed to be highly effective in discriminating between keratoconic and normal eyes [12]. The best fit sphere (BFS) reference body has traditionally been used in elevation maps; nevertheless, there is growing evidence that a toric ellipsoid would be a more useful reference body [12–15]. Given the different toric ellipsoid options available in Scheimpflug tomography, more studies are needed to determine which is the best between the best fit toric ellipsoid (BFTE) and best fit toric ellipsoid with fixed eccentricity (BFTEFE).

To the best of our knowledge, only one study has performed this same comparison [12]. Other existing research studies in the field have used the BFTE as the reference body [13–15]. No previous studies assessing the toric ellipsoid reference body have separated their study populations according to refraction, and it has been demonstrated that myopic and hyperopic populations have different normative data as seen on elevation maps [16, 17]. In light of these issues, the present study aims to establish which reference body offers the best sensitivity, obtain normative data for the myopic population using toric ellipsoid reference bodies, and establish cutoff points for the keratoconus population.

## 2. Materials and Methods

A retrospective, observational study was conducted at the Fundación Jiménez Díaz in Madrid. The study protocols used were in adherence to the tenets of the Declaration of Helsinki. No informed consent was retrieved, as the study was retrospective and participant identities cannot be derived from published data. Local institutional review board approval was obtained prior to data collection. The cornea unit at our institution has a Scheimpflug tomographer (OCULUS Pentacam HD®), and the entire tomographic database was reviewed to obtain the sample of normal and keratoconic myopic eyes.

General inclusion criteria for all eyes (both normal and keratoconus) were as follows: findings on corneal tomography with a quality specification (QS) of “OK” (indicating best possible quality, in which the measurement is correct and reproducible), with corneal coverage of at least 9 mm and no extrapolated data in the central 8 mm. All eyes were required to have simple or compound myopia (mixed astigmatism was excluded). All patients were 18 years of age or older, had not used contact lenses prior to tomography measurements for at least 1 week for soft lenses and 2 weeks for hard lenses. None had a history of corneal surgery or disease that could alter the corneal shape, such as scars, pterygium, and nodules.

Further specific inclusion criteria for normal patients were absence of abnormal findings on biomicroscopy, best-corrected visual acuity of 1.0 on the decimal scale, pachymetry within 475 *μ*m and 650 *μ*m, and no personal or family history of ectasia.

Additional inclusion criteria for keratoconus eyes were the following: abnormal posterior elevation according to the global consensus on keratoconus and ectatic diseases and at least 2 other topographic alterations compatible with keratoconus (corneal thickness spatial profile, percentage thickness increase, inferior/superior index, maximum Ambrosio's relational thickness index, etc.) [18–21]. After selecting keratoconus patients, only those meeting criteria for Stage 1 of the Amsler-Krumeich classification were included.

The patient's age and eyes were recorded. Tomographic data included the root mean square of elevation values for the posterior corneal surface (RMS-P). The elevation of the anterior corneal surface and posterior corneal surface was measured at the following points: anterior elevation at the apex (AA), posterior elevation at the apex (PA), anterior elevation at the thinnest point (AT), and posterior elevation at the thinnest point (PT) using 3 different reference bodies (i.e., BFS, BFTE, and BFTEFE). The elevation map was set in the “float,” “optimise shift,” and manual mode with fixed 8 mm diameter. All reference bodies included are the standard options available in the “front” and “back” elevation maps display. No enhanced reference bodies such as the Belin/Ambrósio enhanced ectasia display (BAD-D) were used.

For the anterior surface, the BFTEFE uses a fixed eccentricity of +0.47, which is equivalent to an asphericity (*Q* value) of −0.22. For the posterior surface, it establishes a fixed eccentricity of −0.45, equivalent to an asphericity (*Q* value) of −0.20. This corresponds to the mean value of the population in the 8 mm zone. The BFTE does not have a fixed eccentricity but rather calculates it each time to best fit the eye studied.

Measuring points are corneal positions chosen to facilitate replication of the measurement. This is why the corneal apex as well as the thinnest point was selected, where pathologic changes are most likely to occur. On the other hand, the RMS is simply a different way to calculate the average of a set of measurements. The reason to use the RMS, and not the more familiar mean, is that when data analyzed have both positive and negative values (like elevation maps have), negative values cancel out positive values when they are added while calculating the mean (e.g., the mean of two elevation values such as +5 *μ*m and −5 *μ*m would give a result of 0 *μ*m, and an average elevation of 0 *μ*m would suggest no elevation, which is not the case). The RMS, instead, calculates the average in a different way. By calculating the square of each value and then the square root of it, all measures end up being positive even if the initial value was of negative sign (overcoming the problem of having positive and negative values). Using the previous example, the square of −5 *μ*m is +25 and the square root of +25 is +5. This way, the RMS of two elevation values like +5 *μ*m and −5 *μ*m would result in 5 *μ*m, meaning that the average elevation value for this cornea is 5 *μ*m away from the reference body. As all values are turned into a positive sign, the result does not indicate whether the value given is above or below the reference body and it only provides information on its distance from the reference body. The RMS can be viewed by right-clicking on the upper part of the elevation maps.

The following information was calculated for all parameters: mean, median, standard deviation, and the percentiles 2.5, 5, 95, and 97.5. The groups were analyzed for normality using the Shapiro–Wilk test. Student's *t*-test was used to determine the presence of statistically significant differences between the normal and keratoconic population at each point of measurement. Receiver operating characteristic (ROC) curves were obtained, and the best cutoff values were calculated according to the Youden index, searching for maximum potential effectiveness by combining sensitivity and specificity.

## 3. Results

A search was done of all the entries contained in the tomographic database at the Fundación Jiménez Díaz hospital from January 2009 until March 2016. Charts and tomographies from a total of 3638 patients were studied. A total of 301 eyes met the inclusion and exclusion criteria, of which 219 were normal myopic eyes and 82 were myopic keratoconic eyes.  Table 1 contains the basic demographic data of the patients studied. The most frequent reasons for exclusion were the failure to meet tomographic quality standards, past surgery, use of contact lenses, and other diseases of the cornea.

Anterior and posterior elevation measurements were taken at the apex and thinnest point, and RMS-P was recorded, using the 3 different reference bodies. Mean, median, standard deviation, and percentile data are summarized in Table 2. Although mean and standard deviation were calculated, the groups studied did not show a normal distribution according to the Shapiro–Wilk test, and therefore, median and percentile values are the correct measures of central tendency and dispersion to assess this population. All points measured show statistically significant difference between the normal and keratoconic population, with the exception of the AA and PA when measured with the BFTE. Toric ellipsoid bodies showed a closer fit. Three out of 4 measurements taken of the normal population using the BFTEFE had a median of 0 (AA, AT, and PT). BFTE had 2 out of 4 measurements with a median of 0 (AA and AT), and BFS had none. For the normal population, the 97.5 percentile of the measures of posterior elevation at the thinnest point was 15.6 *μ*m for BFS, 6.55 *μ*m for BFTEFE, and 4 *μ*m for BFTE.

The normal myopic population was compared to the keratoconic myopic population to determine the best cutoff point to discriminate normal from diseased eyes.  Table 3 shows the cutoff points obtained according to the maximum potential effectiveness. The sensitivity, specificity, positive predictive value (PPV), and negative predictive value (NPV) results obtained, when these cutoff points were used, are shown in the same table. Values obtained at the corneal apex show the worst performance.

Measurements taken at the thinnest point and RMS-P showed the best results; of these, BFS and BFTEFE had superior outcomes at the thinnest point of the posterior elevation map. Cutoff values at this position were 17.5 *μ*m for BFS, 8 *μ*m for BFTEFE, and 3.5 *μ*m for BFTE. Comparing all measurement points from all reference bodies, the best sensitivity (100%) was achieved with the BFTEFE in the PT. The second most sensitive measurement was the BFS in the PT, with a sensitivity of 97.6%. The BFTE had the best performance with the RMS-P, with a sensitivity of 92.7% at a cutoff point of 5.87 *μ*m.

Though this study did not include a subclinical keratoconus population, data from normal myopic population can be used to identify suspicious cases. As the BFTEFE at the PT was the measurement with the greatest degree of sensitivity, it is interesting to outline the percentile associated with the cutoff point of 5 *μ*m (p95) and 6.55 *μ*m (p97.5). An additional calculation was made at a cutoff point of 6 *μ*m, showing this to be p97.

**Figure 1:**
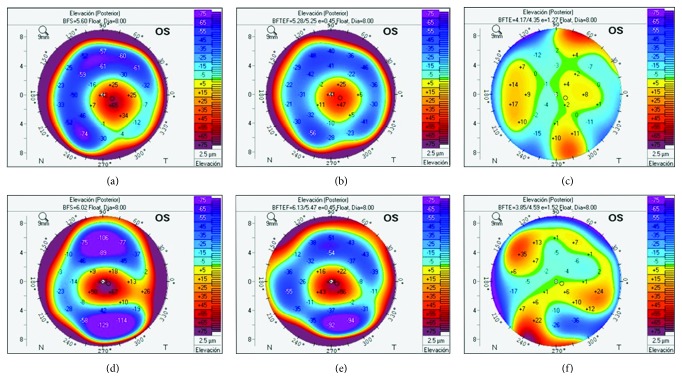
(a–c) The posterior elevation of a patient measured with the BFS (a), BFTEFE (b), and BFTE (c). It is quite evident how, in certain occasions, the BFTE can hide the cone. (d–f) The same problem in a different patient.

Elevation data are obtained manually, meaning that the 3 reference bodies had to be individually visualized and changed in each patient. During this process, it was noticed that in some patients, the BFTE completely hid the cone while the BFS and BFTEFE did not. Two examples of this behavior are shown in Figure 1.

## 4. Discussion

The prevalence of keratoconus varies by region and has been reported to be as low as 0.0003% in a Russian population and up to 4% in an Iranian location [22, 23]. In the United States (US), prevalence has been reported around 0.05% [24]. When prevalence is analyzed in the setting of refractive surgery candidates, percentages are consistently above the general population, with reports of 6.4% [25], 8.59% [26], and up to 24% [27]. Furthermore, it is estimated that over 11 million LASIK procedures were performed in the US by 2011 [28]. This becomes relevant as these procedures have demonstrated a high risk of developing a postsurgical ectasia in the keratoconus population [5–8]. It is therefore clear that although the percentage of iatrogenic ectasia may be considered low, the high volume of surgeries performed makes it an unacceptable frequent encounter in corneal clinics [29, 30]. Consequently, screening processes must continuously search for the most sensitive diagnostic tools.

Elevation maps can be used to detect posterior elevation, that is, one of the earliest signs of keratoconus [9–11]. These maps compare a patient's cornea to a reference body, performing the calculation of the reference body at each individual exam to best fit the studied cornea, outlining the differences in both [31]. Historically, most ophthalmologists are most familiar with the BFS, as it is the one with the most available data, and several tools have been developed based on this reference surface [10, 32, 33]. Doubt has been cast over the toric ellipsoid reference body due to the risk of masking the cone [33]. Nevertheless, recent studies have revisited this option, finding advantages in terms of sensitivity [12–15]. The ideal reference body should be one that most closely resembles the studied cornea to be able to detect early variations from normality while avoiding an almost perfect fit in the cone of pathologic corneas; as such a close fit would mask the cone. The results of our study show that the reference body that best fits this description is the toric ellipsoid with fixed eccentricity.

Corneas are not perfect spheres but rather are prolate and toric [34]. This is why toric ellipsoid reference bodies couple better with the studied cornea than the sphere does. This is evidenced by looking at the measures of central tendency of the BFTEFE and BFTE, which are closer to 0 than those obtained by the BFS. Also, dispersion measurements of the BFS show a wider range compared to both toric ellipsoid bodies, meaning a bigger difference between the reference body and the cornea. Among the toric ellipsoid bodies, the tomographer offers 2 options: toric ellipsoid and toric ellipsoid with fixed eccentricity. To understand the difference between both, it is important to understand what eccentricity means. Eccentricity is a measurement that shows how much an ellipse differs from a circle. Ellipses must have an eccentricity value above 0 (0 is a circle) and less than 1 (1 is a parabola). Fixed eccentricity is the key factor, as it prevents this reference body from creating a nearly exact match, which would mask the cone, while providing a comparative surface that best resembles a normal corneal shape, enabling the early detection of differences. When the option with no fixed eccentricity was used (BFTE), we found several cases in which the reference body masked the cone (Figure 1). This might partly explain the lower sensitivity of the BFTE, and we do not recommend this option. The BFS does not mask the cone and behaves well but, as explained before, the cornea is not a sphere, which is why the sensitivity is not as good as the one obtained with a BFTEFE. Of all the parameters measured, the best results were achieved when measuring posterior elevation at the thinnest point with the BFTEFE, obtaining a sensitivity of 100% when a cutoff point of 8 was used. It is also important to note that this sensitivity is not obtained by sacrificing specificity, as it is near 100% (0.995) as well. BFTEFE consistently showed better performance for all parameters, including PPV and NPV. It is nonetheless important to point out that for KC diagnosis, clinicians should not rely on one single parameter but rather a combination of them. It also must be borne in mind that this study did not include a usual elevation measurement other studies have used such as maximum elevation. This measurement poses a special problem: in cases of high astigmatism, normal patients would show a high elevation value, falsely indicating disease [35]. This does not happen when the measurement is taken at the thinnest point.

These results confirm those obtained by Sideroudi et al. [12], the only difference being that their cutoff point was 7 *μ*m rather than 8 *μ*m as in our study. Though the difference was slight, our groups were not exactly the same, as our research was carried out in a myopic population, and their study did not discriminate according to refraction. Though being a parameter with very good performance, RMS-P does not offer better sensitivity than posterior elevation at the thinnest point. To the best of our knowledge, ours is the first study to assess the cutoff values in a normal and keratoconic myopic population for toric ellipsoid reference bodies.

To arrive at a cutoff point for suspicious cases (a population not included in the study), normative data from the normal population can be used. It should be taken into account that only 5% of the normal population would have 5 *μ*m or more of posterior elevation measured at the PT with the BFTEFE. Using a cutoff point of 6 *μ*m, it would be 3% of the normal population. Depending on the intentions of screening and whether more or less sensitivity is desired, either of these two values would be useful.

Actual color scales for elevation maps in the Pentacam® are designed to highlight pathologic elevation using “hot colors” based on the BFS range. When using a BFTEFE, these same color scales become less intuitive as the range of values with this reference body is lower and, as a result, “hot colors” would only start to appear in more advanced cases. A suggestion would therefore be to include another option in the color scale to change the step width every 1.5 *μ*m (the actual minimal increase is 2.5 *μ*m). By creating this new option, ophthalmologists would be able to view a similarly intuitive image to what they are used to.

As this is a retrospective study, certain sources of bias are expected. First, a lack of data impeded many patients from being included. Second, factors that alter corneal tomography (contact-lens use, scars) may not have been recorded in the charts ending up with the inclusion of patients that should have been excluded. Nevertheless, since all reference bodies were tested in all subjects, we expect this bias to affect the population equally. Third, the most significant difficulty and probable source of bias is the lack of definition for keratoconus within the scientific community. Difficulties in establishing universally accepted diagnostic criteria became evident in the global consensus on keratoconus and ectatic disease [18]. Studies involving this population use different inclusion criteria. Including patients based on tomographic parameters in a study testing some of these same parameters was the biggest challenge as not to bias the sample in favor of one of the reference bodies tested, specially the BFS, as it is the reference body historically used in our cornea service and therefore the one that the investigators are familiar with. Using clinical signs was not an option, as they are not necessarily present in the earliest phases of the disease, which was our targeted population. The strategy to overcome this was not to assume a specific value for pathologic posterior elevation while screening but to allow values surrounding previously suggested abnormal measures in combination with other clear pathologic tomographic alterations that did not rely on posterior elevation.

This study assessed the role of different reference bodies in the diagnosis of keratoconus. Detecting progression is another field in which much has been done but there is still no consensus. Maximum keratometry (Kmax) is probably the most widely used parameter but is not yet the ideal one. The usual cutoff has been set in a 1-diopter increase in 1 year. However, variations of up to 1.34 diopters can be obtained in the same patient on exams taken on the same day, especially in advanced keratoconus [36]. Given the more accurate fit of the BFTEFE, it could be hypothesized that it would be a good tool to detect progression. Studies will be needed to prove this.

On a similar matter, novel techniques have been introduced in the search for the most sensitive tool. Ambrosio developed an index integrating corneal biomechanics and tomographic data, showing promising results [37]. Studies undertaken in this area have most often used and compared biomechanics to sphere-based reference bodies, especially the BAD-D [38–42]. It would be of interest to know how the BFTEFE behaves against these parameters and whether its integration would enhance detection.

## 5. Conclusions

BFTEFE outperformed the BFS and BFTE in diagnosing KC. Of the different measuring points studied, the greatest sensitivity when differentiating between the normal and KC population was achieved by posterior elevation measured at the thinnest point with the BFTEFE, using a cutoff point of 8 *μ*m. The BFTE was found to hide the cone in certain patients and, therefore, should be considered unreliable for KC screening.

## Figures and Tables

**Table 1:** Demographic data.

Parameter	Group	
	KC myopic (*n* = 82 eyes)	Normal myopic (*n* = 219 eyes)
Age, median	37 (IQR 23–57)	33 (IQR 22–55)
Sex		
Male	64.6%	46.1%
Female	35.4%	53.9%
Eye		
Right	52.4%	45.2%
Left	47.6%	54.8%

KC, keratoconus; IQR, interquartile range.

**Table 2:** Mean, median, standard deviation, and percentiles for each measurement point in the normal and keratoconic population.

Parameter	Group	Mean	SD	Median	Percentiles				*P*
					2.5%	5%	95%	97.5%	
BFS	KC myopic	29.0	8.34	27.1	15.3	17.5	42.0	44.5	<0.001
RMS-P	Normal myopic	15.1	6.97	13.7	5.33	5.91	28.2	31.5	

BFS AA	KC myopic	5.02	4.68	4.00	−2.00	0.00	14.9	18.0	<0.001
	Normal myopic	1.80	1.11	2.00	0.00	0.00	4.00	4.00	

BFS PA	KC myopic	13.3	12.8	12.0	−10.9	−3.00	32.0	35.9	<0.001
	Normal myopic	1.96	3.06	2.00	−4.00	−3.00	8.0	8.0	

BFS AT	KC myopic	13.0	5.80	12.0	5.00	6.00	23.0	27.0	<0.001
	Normal myopic	2.21	1.71	2.00	−0.55	0.00	5.00	5.55	

BFS PT	KC myopic	34.7	12.7	32.0	18.0	19.0	53.0	70.8	<0.001
	Normal myopic	4.81	4.31	4.00	−2.55	−1.00	13.0	15.6	

BFTEFE	KC myopic	13.6	4.73	12.4	6.40	8.14	22.2	25.0	<0.001
RMS-P	Normal myopic	4.20	1.35	4.07	2.36	2.50	6.84	7.60	

BFTEFE	KC myopic	3.51	4.63	3.00	−2.98	−2.00	13.0	16.9	<0.001
AA	Normal myopic	−0.27	1.07	0.00	−2.00	−2.00	1.00	2.00	

BFTEFE	KC myopic	10.3	12.6	9.50	−12.9	−6.00	29.9	33.9	<0.001
PA	Normal myopic	−1.90	3.18	−2.00	−8.00	−7.00	4.00	4.55	

BFTEFE	KC myopic	7.96	5.48	6.00	1.02	2.00	20.0	21.0	<0.001
AT	Normal myopic	−0.21	0.93	0.00	−2.00	−2.00	1.00	1.00	

BFTEFE	KC myopic	24.4	12.0	22.5	9.00	10.0	45.8	57.8	<0.001
PT	Normal myopic	0.07	2.80	0.00	−5.00	−4.10	5.00	6.55	

BFTE	KC myopic	10.2	3.79	9.45	4.27	4.92	17.1	18.1	<0.001
RMS-P	Normal myopic	3.84	1.09	3.73	2.20	2.34	6.03	6.33	

BFTE AA	KC myopic	−0.79	2.23	−1.00	−4.97	−4.00	3.00	4.97	0.469
	Normal myopic	−0.35	0.67	0.00	−1.00	−1.00	1.00	1.00	

BFTE PA	KC myopic	−1.61	5.16	−1.00	−11.0	−9.00	5.95	6.00	0.068
	Normal myopic	−3.11	1.44	−3.00	−6.00	−6.00	−1.00	0.00	

BFTE AT	KC myopic	3.15	3.10	3.00	−1.98	0.00	8.90	11.0	<0.001
	Normal myopic	−0.29	0.69	0.00	−2.00	−1.00	1.00	1.00	

BFTE PT	KC myopic	10.5	7.41	9.00	−1.00	−0.95	21.0	24.0	<0.001
	Normal myopic	−1.05	2.40	−1.00	−5.00	−5.00	3.00	4.00	

AA, anterior elevation at the apex; AT, anterior elevation at the thinnest point; BFS, best fit sphere; BFTE, best fit toric ellipsoid; BFTEFE, best fit toric ellipsoid with fixed eccentricity; KC, keratoconus; RMS-P, root mean square of posterior elevations; SD, standard deviation; PA, posterior elevation at the apex; PT, posterior elevation at the thinnest point.

**Table 3:** Cutoff values between normal and keratoconus population at each measuring point with the statistical measures obtained at these cutoff values.

Parameter	Cutoff	Sensitivity	Specificity	PPV	NPV	AUC (95% CI)
BFS RMS-P	18.7	0.939	0.763	0.597	0.971	0.900 (0.870, 0.940)
BFS AA	3.50	0.585	0.941	0.787	0.858	0.760 (0.680, 0.840)
BFS PA	7.50	0.744	0.945	0.836	0.908	0.840 (0.770, 0.910)
BFS AT	5.50	0.951	0.973	0.929	0.982	0.990 (0.990, 1.000)
BFS PT	17.5	0.976	0.991	0.976	0.991	1.000 (1.000, 1.000)
BFTEFE RMS-P	7.99	0.951	0.982	0.951	0.982	0.990 (0.990, 1.000)
BFTEFE AA	1.50	0.659	0.968	0.885	0.883	0.800 (0.730, 0.870)
BFTEFE PA	4.50	0.732	0.973	0.909	0.906	0.860 (0.790, 0.920)
BFTEFE AT	1.50	0.963	0.977	0.940	0.986	0.980 (0.950, 1.000)
BFTEFE PT	8.00	1.000	0.995	0.988	1.000	1.000 (1.000, 1.000)
BFTE RMS-P	5.87	0.927	0.945	0.864	0.972	0.980 (0.960, 1.000)
BFTE AA	−1.50	0.402	0.977	0.868	0.814	0.590 (0.510, 0.680)
BFTE PA	−1.50	0.512	0.900	0.656	0.831	0.620 (0.520, 0.710)
BFTE AT	0.50	0.841	0.932	0.821	0.940	0.910 (0.860, 0.960)
BFTE PT	3.50	0.866	0.968	0.910	0.951	0.950 (0.920, 0.990)

AA, anterior elevation at the apex; AT, anterior elevation at the thinnest point; AUC, area under the curve; BFS, best fit sphere; BFTE, best fit toric ellipsoid; BFTEFE, best fit toric ellipsoid with fixed eccentricity; CI, confidence interval; NPV, negative predictive value; RMS-P, root mean square of posterior elevations; PA, posterior elevation at the apex; PPV, positive predictive value; PT, posterior elevation at the thinnest point.

## Data Availability

The data used to support the findings of this study are included within the article.
